# 1,5-Dimethyl-2-nitro­imino-1,3,5-tri­azinane

**DOI:** 10.1107/S1600536808021533

**Published:** 2008-07-16

**Authors:** Cong Zhao, Wen-ge Yang, Yong-hong Hu, Lei Shen, Xiu-tao Lu

**Affiliations:** a College of Life Science and Pharmaceutical Engineering, Nanjing University of Technology, Xinmofan Road No. 5, Nanjing 210009, People’s Republic of China

## Abstract

The asymmetric unit of the title compound, C_5_H_11_N_5_O_2_, contains two independent mol­ecules. The two triazine rings adopt envelope conformations. Intra­molecular C—H⋯N and N—H⋯O hydrogen bonds result in the formation of two five- and two six-membered rings which are nearly planar; in addition, they are also nearly coplanar. In the crystal structure, inter­molecular N—H⋯N, C—H⋯N and C—H⋯O hydrogen bonds link the mol­ecules.

## Related literature

For general background, see: Wakita *et al.* (2003 ▶). For related literature, see: Shiokawa *et al.* (1991 ▶).
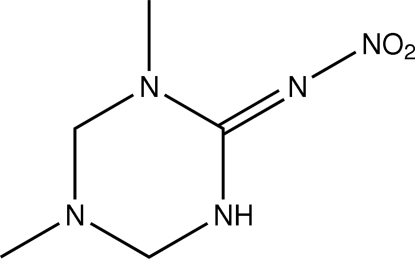


## Experimental

### 

#### Crystal data

C_5_H_11_N_5_O_2_
*M* *_r_* = 173.19
Monoclinic, 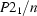

*a* = 6.6490 (13) Å
*b* = 30.103 (6) Å
*c* = 8.2940 (17) Å
β = 104.19 (3)°
*V* = 1609.4 (6) Å^3^
*Z* = 8
Mo *K*α radiation
μ = 0.11 mm^−1^
*T* = 294 (2) K
0.30 × 0.10 × 0.10 mm

#### Data collection

Enraf–Nonius CAD-4 diffractometer
Absorption correction: ψ scan (North *et al.*, 1968 ▶) *T*_min_ = 0.967, *T*_max_ = 0.989
3126 measured reflections
2873 independent reflections
1979 reflections with *I* > 2σ(*I*)
*R*_int_ = 0.037
3 standard reflections frequency: 120 min intensity decay: none

#### Refinement

*R*[*F* ^2^ > 2σ(*F* ^2^)] = 0.072
*wR*(*F* ^2^) = 0.194
*S* = 1.00
2873 reflections
217 parameters
H-atom parameters constrained
Δρ_max_ = 0.36 e Å^−3^
Δρ_min_ = −0.38 e Å^−3^

### 

Data collection: *CAD-4 Software* (Enraf–Nonius, 1989 ▶); cell refinement: *CAD-4 Software*; data reduction: *XCAD4* (Harms & Wocadlo, 1995 ▶); program(s) used to solve structure: *SHELXS97* (Sheldrick, 2008 ▶); program(s) used to refine structure: *SHELXL97* (Sheldrick, 2008 ▶); molecular graphics: *PLATON* (Spek, 2003 ▶); software used to prepare material for publication: *SHELXTL* (Sheldrick, 2008 ▶).

## Supplementary Material

Crystal structure: contains datablocks global, I. DOI: 10.1107/S1600536808021533/hk2491sup1.cif

Structure factors: contains datablocks I. DOI: 10.1107/S1600536808021533/hk2491Isup2.hkl

Additional supplementary materials:  crystallographic information; 3D view; checkCIF report

## Figures and Tables

**Table 1:** Hydrogen-bond geometry (Å, °)

*D*—H⋯*A*	*D*—H	H⋯*A*	*D*⋯*A*	*D*—H⋯*A*
N2—H2*A*⋯O1	0.86	1.94	2.549 (5)	126
N7—H7*A*⋯O3	0.86	1.99	2.583 (5)	126
N7—H7*A*⋯N3^i^	0.86	2.57	3.210 (5)	132
C1—H1*C*⋯O3^ii^	0.96	2.54	3.309 (6)	137
C2—H2*B*⋯N4	0.96	2.24	2.699 (6)	108
C4—H4*B*⋯O4^iii^	0.97	2.50	3.411 (6)	156
C4—H4*C*⋯O3^ii^	0.97	2.49	3.251 (6)	136
C7—H7*B*⋯N9	0.96	2.21	2.670 (5)	108
C7—H7*B*⋯O4^iv^	0.96	2.59	3.317 (6)	133
C8—H8*B*⋯O3^iii^	0.97	2.56	3.420 (5)	148
C9—H9*C*⋯O1^v^	0.97	2.59	3.359 (6)	137

Symmetry codes: (i) 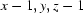; (ii) 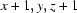; (iii) 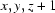; (iv) 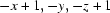; (v) 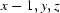.

**Fig. 1.**
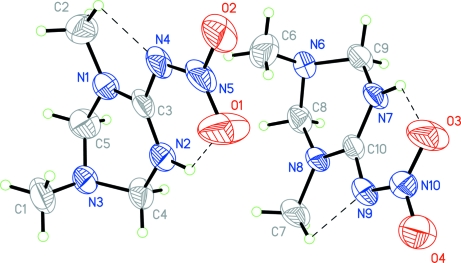
The molecular structure of the title molecule, with the atom-numbering scheme. Displacement ellipsoids are drawn at the 30% probability level. Hydrogen bonds are shown as dashed lines.

## supplementary crystallographic information

### Comment

Nitroguanidine derivatives have a high insecticidal activity and a wide spectrum (Wakita *et al.*,  2003). As part of our ongoing studies in this area, we report herein the crystal structure of the title compound.

The asymmetric unit of the title compound (Fig. 1) contains two independent molecules, in which the bond lengths and angles are generally within normal ranges. Rings A (N1-N3/C3-C5) and B (N6-N8/C8-C10) have envelope conformations, with N3 and N6 atoms displaced by -0.652 (2) and -0.645 (3) Å, respectively, from the plane of the other rings atoms. The intramolecular C-H···N and N-H···O hydrogen bonds (Table 1) result in the formation of nearly planar two five- and two six-membered rings: C (N1/N4/C2/C3/H2B), D (O1/N2/N4/N5/C3/H2A) and E (N8/N9/C7/C10/H7B), F (O3/N7/N9/N10/C10/H7A). The dihedral angles between the rings are C/D = 1.63 (3)° and E/F = 3.43 (3)°. So, rings C, D and E, F are nearly coplanar.

In the crystal structure, intermolecular N-H···N, C-H···N and C-H···O hydrogen bonds (Table 1) link the molecules, in which they may be effective in the stabilization of the structure.

### Experimental

The title compound was synthesized according to the literature method (Shiokawa *et al.*,  1991). Crystals suitable for X-ray analysis were obtained by slow evaporation of an ethanol solution.

### Refinement

H atoms were positioned geometrically, with N-H = 0.86 Å (for NH) and C-H = 0.97 and 0.96 Å for methylene and methyl H, respectively, and constrained to ride on their parent atoms with U_iso_(H) = xU_eq_(C,N), where x = 1.5 for methyl H and x = 1.2 for all other H atoms.

### Crystal data

**Table:**

C_5_H_11_N_5_O_2_	*F*_000_ = 736
*M**_r_* = 173.19	*D*_x_ = 1.430 Mg m^−^^3^
Monoclinic, *P*2_1_/*n*	Mo *K*α radiation λ = 0.71073 Å
Hall symbol: -P 2yn	Cell parameters from 25 reflections
*a* = 6.6490 (13) Å	θ = 9–12º
*b* = 30.103 (6) Å	µ = 0.11 mm^−^^1^
*c* = 8.2940 (17) Å	*T* = 294 (2) K
β = 104.19 (3)º	Block, colorless
*V* = 1609.4 (6) Å^3^	0.30 × 0.10 × 0.10 mm
*Z* = 8	

### Data collection

**Table:**

Enraf–Nonius CAD-4 diffractometer	*R*_int_ = 0.037
Radiation source: fine-focus sealed tube	θ_max_ = 25.2º
Monochromator: graphite	θ_min_ = 1.4º
*T* = 294(2) K	*h* = −7→7
ω/2θ scans	*k* = 0→36
Absorption correction: ψ scan(North *et al.*, 1968)	*l* = 0→9
*T*_min_ = 0.967, *T*_max_ = 0.989	3 standard reflections
3126 measured reflections	every 120 min
2873 independent reflections	intensity decay: none
1979 reflections with *I* > 2σ(*I*)	

### Refinement

**Table:**

Refinement on *F*^2^	Secondary atom site location: difference Fourier map
Least-squares matrix: full	Hydrogen site location: inferred from neighbouring sites
*R*[*F*^2^ > 2σ(*F*^2^)] = 0.072	H-atom parameters constrained
*wR*(*F*^2^) = 0.194	*w* = 1/[σ^2^(*F*_o_^2^) + (0.06*P*)^2^ + 4*P*] where *P* = (*F*_o_^2^ + 2*F*_c_^2^)/3
*S* = 1.00	(Δ/σ)_max_ < 0.001
2873 reflections	Δρ_max_ = 0.36 e Å^−^^3^
217 parameters	Δρ_min_ = −0.37 e Å^−^^3^
Primary atom site location: structure-invariant direct methods	Extinction correction: none

### Special details

**Table:**

Geometry. All e.s.d.'s (except the e.s.d. in the dihedral angle between two l.s. planes) are estimated using the full covariance matrix. The cell e.s.d.'s are taken into account individually in the estimation of e.s.d.'s in distances, angles and torsion angles; correlations between e.s.d.'s in cell parameters are only used when they are defined by crystal symmetry. An approximate (isotropic) treatment of cell e.s.d.'s is used for estimating e.s.d.'s involving l.s. planes.
Refinement. Refinement of F^2^ against ALL reflections. The weighted R-factor wR and goodness of fit S are based on F^2^, conventional R-factors R are based on F, with F set to zero for negative F^2^. The threshold expression of F^2^ > 2sigma(F^2^) is used only for calculating R-factors(gt) etc. and is not relevant to the choice of reflections for refinement. R-factors based on F^2^ are statistically about twice as large as those based on F, and R- factors based on ALL data will be even larger.

### Fractional atomic coordinates and isotropic or equivalent isotropic displacement parameters (Å^2^)

**Table:**

	*x*	*y*	*z*	*U*_iso_*/*U*_eq_	
O1	0.4157 (7)	0.16494 (15)	0.6298 (4)	0.0982 (15)	
O2	0.2294 (5)	0.22215 (11)	0.5490 (4)	0.0699 (10)	
N1	0.3763 (5)	0.19429 (11)	1.0927 (4)	0.0447 (8)	
N2	0.4955 (5)	0.14315 (11)	0.9359 (4)	0.0466 (8)	
H2A	0.5027	0.1334	0.8400	0.056*	
N3	0.6537 (5)	0.14525 (12)	1.2299 (4)	0.0501 (9)	
N4	0.3096 (5)	0.20872 (11)	0.8150 (4)	0.0469 (8)	
N5	0.3202 (5)	0.19792 (13)	0.6628 (4)	0.0549 (10)	
C1	0.8312 (7)	0.17403 (17)	1.2213 (7)	0.0731 (15)	
H1A	0.8666	0.1925	1.3184	0.110*	
H1B	0.7939	0.1924	1.1238	0.110*	
H1C	0.9481	0.1559	1.2160	0.110*	
C2	0.2708 (8)	0.23592 (15)	1.1160 (6)	0.0616 (12)	
H2B	0.2130	0.2493	1.0097	0.092*	
H2C	0.3688	0.2559	1.1835	0.092*	
H2D	0.1617	0.2297	1.1701	0.092*	
C3	0.3970 (6)	0.18117 (13)	0.9446 (5)	0.0442 (9)	
C4	0.5922 (7)	0.11728 (14)	1.0841 (5)	0.0500 (10)	
H4B	0.4951	0.0950	1.1029	0.060*	
H4C	0.7132	0.1020	1.0657	0.060*	
C5	0.4757 (8)	0.16943 (17)	1.2470 (5)	0.0621 (13)	
H5A	0.5162	0.1902	1.3387	0.075*	
H5B	0.3757	0.1489	1.2734	0.075*	
O3	0.0455 (5)	0.07427 (13)	0.2450 (4)	0.0804 (12)	
O4	0.3371 (5)	0.04202 (11)	0.2690 (4)	0.0640 (9)	
N6	−0.1841 (5)	0.09106 (11)	0.7596 (4)	0.0466 (8)	
N7	−0.1239 (4)	0.07164 (11)	0.4938 (4)	0.0421 (8)	
H7A	−0.1508	0.0788	0.3904	0.051*	
N8	0.0994 (5)	0.04358 (11)	0.7263 (4)	0.0429 (8)	
N9	0.2160 (5)	0.04411 (11)	0.4922 (4)	0.0424 (8)	
N10	0.1942 (5)	0.05374 (11)	0.3332 (4)	0.0426 (8)	
C6	−0.0729 (8)	0.13372 (16)	0.7715 (7)	0.0729 (15)	
H6A	−0.0136	0.1407	0.8863	0.109*	
H6B	−0.1680	0.1567	0.7221	0.109*	
H6C	0.0355	0.1316	0.7138	0.109*	
C7	0.2916 (7)	0.02129 (16)	0.8125 (5)	0.0579 (12)	
H7B	0.3745	0.0154	0.7351	0.087*	
H7C	0.2587	−0.0062	0.8589	0.087*	
H7D	0.3674	0.0401	0.9001	0.087*	
C8	−0.0443 (6)	0.05545 (15)	0.8268 (5)	0.0486 (10)	
H8A	−0.1253	0.0294	0.8391	0.058*	
H8B	0.0352	0.0639	0.9369	0.058*	
C9	−0.2774 (6)	0.07940 (15)	0.5863 (5)	0.0481 (10)	
H9B	−0.3612	0.0529	0.5830	0.058*	
H9C	−0.3682	0.1033	0.5342	0.058*	
C10	0.0585 (5)	0.05359 (12)	0.5651 (4)	0.0371 (8)	

### Atomic displacement parameters (Å^2^)

**Table:**

	*U*^11^	*U*^22^	*U*^33^	*U*^12^	*U*^13^	*U*^23^
O1	0.131 (3)	0.121 (3)	0.0415 (19)	0.080 (3)	0.019 (2)	0.023 (2)
O2	0.085 (2)	0.070 (2)	0.0385 (16)	0.0103 (18)	−0.0163 (15)	0.0154 (15)
N1	0.0489 (19)	0.050 (2)	0.0296 (16)	0.0062 (15)	−0.0016 (14)	0.0029 (14)
N2	0.053 (2)	0.050 (2)	0.0286 (16)	0.0079 (16)	−0.0075 (14)	0.0035 (14)
N3	0.054 (2)	0.054 (2)	0.0308 (17)	0.0081 (17)	−0.0110 (14)	0.0006 (15)
N4	0.050 (2)	0.051 (2)	0.0281 (17)	−0.0018 (16)	−0.0123 (14)	0.0093 (14)
N5	0.049 (2)	0.059 (2)	0.043 (2)	0.0020 (18)	−0.0152 (16)	0.0148 (18)
C1	0.056 (3)	0.058 (3)	0.082 (4)	−0.009 (2)	−0.026 (3)	−0.001 (3)
C2	0.068 (3)	0.058 (3)	0.055 (3)	0.011 (2)	0.007 (2)	0.002 (2)
C3	0.039 (2)	0.043 (2)	0.039 (2)	−0.0049 (17)	−0.0137 (16)	0.0054 (17)
C4	0.059 (3)	0.044 (2)	0.036 (2)	−0.0027 (19)	−0.0087 (18)	0.0081 (18)
C5	0.075 (3)	0.070 (3)	0.033 (2)	0.017 (3)	−0.004 (2)	0.010 (2)
O3	0.080 (2)	0.117 (3)	0.0414 (17)	0.052 (2)	0.0087 (16)	0.0229 (18)
O4	0.0575 (19)	0.079 (2)	0.0566 (19)	0.0107 (17)	0.0153 (15)	0.0055 (16)
N6	0.0420 (18)	0.051 (2)	0.0409 (18)	−0.0008 (15)	−0.0012 (14)	−0.0055 (16)
N7	0.0349 (17)	0.059 (2)	0.0275 (16)	0.0067 (15)	−0.0026 (13)	0.0045 (14)
N8	0.0411 (18)	0.053 (2)	0.0274 (16)	0.0113 (15)	−0.0043 (13)	0.0025 (14)
N9	0.0380 (17)	0.050 (2)	0.0326 (17)	0.0027 (14)	−0.0036 (13)	0.0066 (14)
N10	0.0430 (18)	0.045 (2)	0.0373 (17)	0.0064 (15)	0.0054 (14)	0.0010 (14)
C6	0.065 (3)	0.057 (3)	0.094 (4)	−0.012 (2)	0.015 (3)	−0.022 (3)
C7	0.054 (3)	0.072 (3)	0.038 (2)	0.016 (2)	−0.0067 (19)	0.012 (2)
C8	0.050 (2)	0.061 (3)	0.031 (2)	0.001 (2)	0.0023 (17)	0.0038 (18)
C9	0.036 (2)	0.064 (3)	0.040 (2)	0.0028 (19)	0.0014 (16)	0.0006 (19)
C10	0.0341 (19)	0.035 (2)	0.0353 (19)	−0.0013 (15)	−0.0040 (15)	−0.0016 (15)

### Geometric parameters (Å, °)

**Table:**

O1—N5	1.245 (5)	O3—N10	1.241 (4)
O2—N5	1.228 (4)	O4—N10	1.249 (4)
N1—C3	1.329 (5)	N6—C8	1.439 (5)
N1—C2	1.472 (5)	N6—C9	1.461 (5)
N1—C5	1.490 (5)	N6—C6	1.473 (6)
N2—C3	1.329 (5)	N7—C10	1.328 (4)
N2—C4	1.464 (5)	N7—C9	1.438 (5)
N2—H2A	0.8600	N7—H7A	0.8600
N3—C5	1.426 (6)	N8—C10	1.332 (5)
N3—C4	1.448 (5)	N8—C8	1.458 (5)
N3—C1	1.480 (6)	N8—C7	1.465 (5)
N4—N5	1.322 (5)	N9—N10	1.323 (4)
N4—C3	1.369 (5)	N9—C10	1.362 (5)
C1—H1A	0.9600	C6—H6A	0.9600
C1—H1B	0.9600	C6—H6B	0.9600
C1—H1C	0.9600	C6—H6C	0.9600
C2—H2B	0.9600	C7—H7B	0.9600
C2—H2C	0.9600	C7—H7C	0.9600
C2—H2D	0.9600	C7—H7D	0.9600
C4—H4B	0.9700	C8—H8A	0.9700
C4—H4C	0.9700	C8—H8B	0.9700
C5—H5A	0.9700	C9—H9B	0.9700
C5—H5B	0.9700	C9—H9C	0.9700
			
C3—N1—C2	122.4 (3)	C8—N6—C9	106.3 (3)
C3—N1—C5	121.3 (3)	C8—N6—C6	110.9 (3)
C2—N1—C5	116.1 (3)	C9—N6—C6	111.1 (4)
C3—N2—C4	122.3 (3)	C10—N7—C9	121.2 (3)
C3—N2—H2A	118.9	C10—N7—H7A	119.4
C4—N2—H2A	118.9	C9—N7—H7A	119.4
C5—N3—C4	108.0 (3)	C10—N8—C8	121.2 (3)
C5—N3—C1	113.4 (4)	C10—N8—C7	122.2 (3)
C4—N3—C1	111.4 (4)	C8—N8—C7	116.6 (3)
N5—N4—C3	119.3 (4)	N10—N9—C10	119.2 (3)
O2—N5—O1	119.1 (4)	O3—N10—O4	118.0 (3)
O2—N5—N4	117.2 (4)	O3—N10—N9	125.0 (3)
O1—N5—N4	123.7 (3)	O4—N10—N9	117.0 (3)
N3—C1—H1A	109.5	N6—C6—H6A	109.5
N3—C1—H1B	109.5	N6—C6—H6B	109.5
H1A—C1—H1B	109.5	H6A—C6—H6B	109.5
N3—C1—H1C	109.5	N6—C6—H6C	109.5
H1A—C1—H1C	109.5	H6A—C6—H6C	109.5
H1B—C1—H1C	109.5	H6B—C6—H6C	109.5
N1—C2—H2B	109.5	N8—C7—H7B	109.5
N1—C2—H2C	109.5	N8—C7—H7C	109.5
H2B—C2—H2C	109.5	H7B—C7—H7C	109.5
N1—C2—H2D	109.5	N8—C7—H7D	109.5
H2B—C2—H2D	109.5	H7B—C7—H7D	109.5
H2C—C2—H2D	109.5	H7C—C7—H7D	109.5
N1—C3—N2	118.0 (3)	N6—C8—N8	114.4 (3)
N1—C3—N4	115.2 (4)	N6—C8—H8A	108.7
N2—C3—N4	126.8 (4)	N8—C8—H8A	108.7
N3—C4—N2	111.6 (3)	N6—C8—H8B	108.7
N3—C4—H4B	109.3	N8—C8—H8B	108.7
N2—C4—H4B	109.3	H8A—C8—H8B	107.6
N3—C4—H4C	109.3	N7—C9—N6	112.2 (3)
N2—C4—H4C	109.3	N7—C9—H9B	109.2
H4B—C4—H4C	108.0	N6—C9—H9B	109.2
N3—C5—N1	112.0 (4)	N7—C9—H9C	109.2
N3—C5—H5A	109.2	N6—C9—H9C	109.2
N1—C5—H5A	109.2	H9B—C9—H9C	107.9
N3—C5—H5B	109.2	N7—C10—N8	118.6 (3)
N1—C5—H5B	109.2	N7—C10—N9	127.3 (3)
H5A—C5—H5B	107.9	N8—C10—N9	114.1 (3)
			
C3—N4—N5—O2	175.8 (4)	C10—N9—N10—O3	5.9 (6)
C3—N4—N5—O1	−4.1 (6)	C10—N9—N10—O4	−175.7 (3)
C2—N1—C3—N2	179.7 (4)	C9—N6—C8—N8	50.4 (4)
C5—N1—C3—N2	5.2 (6)	C6—N6—C8—N8	−70.5 (5)
C2—N1—C3—N4	−1.2 (6)	C10—N8—C8—N6	−21.6 (5)
C5—N1—C3—N4	−175.7 (4)	C7—N8—C8—N6	156.9 (4)
C4—N2—C3—N1	−4.1 (6)	C10—N7—C9—N6	33.9 (5)
C4—N2—C3—N4	176.9 (4)	C8—N6—C9—N7	−56.1 (4)
N5—N4—C3—N1	−179.0 (3)	C6—N6—C9—N7	64.7 (5)
N5—N4—C3—N2	0.0 (6)	C9—N7—C10—N8	−1.5 (5)
C5—N3—C4—N2	55.1 (5)	C9—N7—C10—N9	179.4 (4)
C1—N3—C4—N2	−70.1 (4)	C8—N8—C10—N7	−5.1 (5)
C3—N2—C4—N3	−27.1 (5)	C7—N8—C10—N7	176.4 (4)
C4—N3—C5—N1	−54.2 (5)	C8—N8—C10—N9	174.1 (3)
C1—N3—C5—N1	69.8 (5)	C7—N8—C10—N9	−4.3 (5)
C3—N1—C5—N3	25.4 (6)	N10—N9—C10—N7	0.9 (6)
C2—N1—C5—N3	−149.4 (4)	N10—N9—C10—N8	−178.3 (3)

### Hydrogen-bond geometry (Å, °)

**Table:**

*D*—H···*A*	*D*—H	H···*A*	*D*···*A*	*D*—H···*A*
N2—H2A···O1	0.86	1.94	2.549 (5)	126
N7—H7A···O3	0.86	1.99	2.583 (5)	126
N7—H7A···N3^i^	0.86	2.57	3.210 (5)	132
C1—H1C···O3^ii^	0.96	2.54	3.309 (6)	137
C2—H2B···N4	0.96	2.24	2.699 (6)	108
C4—H4B···O4^iii^	0.97	2.50	3.411 (6)	156
C4—H4C···O3^ii^	0.97	2.49	3.251 (6)	136
C7—H7B···N9	0.96	2.21	2.670 (5)	108
C7—H7B···O4^iv^	0.96	2.59	3.317 (6)	133
C8—H8B···O3^iii^	0.97	2.56	3.420 (5)	148
C9—H9C···O1^v^	0.97	2.59	3.359 (6)	137

Symmetry codes: (i) *x*−1, *y*, *z*−1; (ii) *x*+1, *y*, *z*+1; (iii) *x*, *y*, *z*+1; (iv) −*x*+1, −*y*, −*z*+1; (v) *x*−1, *y*, *z*.
